# Upregulation of long intergenic non-coding RNA LINC00326 inhibits non-small cell lung carcinoma progression by blocking Wnt/β-catenin pathway through modulating the miR-657/dickkopf WNT signaling pathway inhibitor 2 axis

**DOI:** 10.1186/s13062-023-00359-9

**Published:** 2023-02-06

**Authors:** Yingqian Zhang, Jiao Yuan, Mengfei Guo, Run Xiang, Tianpeng Xie, Xiang Zhuang, Wei Dai, Qiang Li, Qi Lai

**Affiliations:** 1 School of Basic Medical Science, Southwest Medical University, Luzhou, 646000 China; 2 Laboratory of Nonhuman Primate Disease Modeling Research, West China Hospital, Sichuan University, Chengdu, 610041 Sichuan China; 3 Department of Respirology and Critical Care Medicine, Chengdu Seventh People’s Hospital, Chengdu, 610041 Sichuan China; 4 Department of Thoracic Surgery, The Affiliated Traditional Chinese Medicine Hospital of Southwest Medical University, Luzhou, 646000 Sichuan China; 5 Department of Thoracic Surgery, Sichuan Cancer Hospital & Institute, Sichuan Cancer Center, School of Medicine, University of Electronic Science and Technology of China, Chengdu, 610000 Sichuan China

**Keywords:** LINC00326, NSCLC, miR-657, DKK2, Wnt/β-catenin pathway

## Abstract

**Background:**

Long intergenic non-coding RNA 326 (LINC00326) modulates hepatocarcinogenic lipid metabolism. However, the ability of LINC00326 to modulate the highly aggressive non-small cell lung carcinoma (NSCLC) is unknown. Here, LINC00326 in NSCLC was investigated, together with its effects on tumor malignancy and the underlying mechanisms of action.

**Methods:**

LINC00326 levels in tumor tissues and cell lines were measured by Real-time fluorescence quantitative polymerase chain reaction (RT-qPCR) and RNA fluorescence in situ hybridization (FISH). Proliferation and apoptosis were assessed in cell lines by Cell Counting Kit-8 (CCK-8), EdU staining assays and flow cytometry, respectively, and tumor growth was measured in mouse models. Possible microRNA targets of LINC00326 were predicted by bioinformatics and verified by RNA pull-down and immunoprecipitation and luciferase reporter assays. Western blotting was used to evaluate the expression of Wnt/β-catenin-associated proteins.

**Results:**

LINC00326 was downregulated in tumor tissues and cell lines. Knockdown of LINC00326 stimulated NSCLC cell proliferation and suppressed apoptosis in vitro*,* as well as enhancing xenograft tumor growth. LINC00326 sponged miR-657, and dickkopf WNT signaling pathway inhibitor 2 (DKK2) was found to be directly targeted by miR-657, with LINC00326 positively regulating its expression through sponging miR-657. The actions of LINC00326 knockdown on proliferation and apoptosis were reversed by stimulation of the miR-657/DKK2 axis. Furthermore, overexpression of miR-657 mitigated DKK2 inhibition on Wnt/β-catenin signaling.

**Conclusions:**

LINC00326/miR-657/DKK2 axis signaling blocked tumor-associated functions in NSCLC cells through the targeting Wnt/β-catenin pathway. This suggests that this pathway could be a target for NSCLC treatment.

**Supplementary Information:**

The online version contains supplementary material available at 10.1186/s13062-023-00359-9.

## Background

Lung cancer is both common and highly aggressive. Non-small cell lung cancer (NSCLC) is the most prevalent form of lung tumor [1, 2], with significant increases in both incidence and mortality seen over the past 50 years [3]. However, despite intensive research into its pathogenesis and treatment options [4], identifying effective interventions has proved elusive [5], indicating the importance of identifying new therapeutic targets.

Since their discovery, non-coding RNAs have received much attention [6]. Long non-coding RNAs (lncRNAs) contain > 200 nucleotides and do not code for protein [7]. LncRNAs are reported to modulate many functions of malignant cells [8–10], often through changes in stimulatory or inhibitory signaling pathways. LncRNAs can also control gene expression, post-transcriptional modifications, chromatin remodeling, and the modulation of translation [11, 12]. Alterations in lncRNA expression and function have been regarded as diagnostic or prognostic markers for NSCLC [13, 14]. Specifically, upregulation of LINC00346 inhibits proliferation of NSCLC cells through mediating JAK-STAT3 signaling pathway [15]. LncRNA HOXA11-AS [16] and lncRNA PINT [17] are associated with advanced clinical stages in NSCLC. LncRNA FENDRR suppresses the progression of NSCLC by regulating the miR-761/TIMP2 axis [18]. Recently, LINC00326 has recently been found to be an important regulator in acrosome biogenesis and vesicle trafficking via targeting G protein subunit beta 5 (GNB5) [19]. Sondergaard et al. [20] showed that LINC00326 modulates lipid metabolism by interacting with the non-canonical RNA-binding protein chaperonin containing TCP1 subunit 3 (CCT3). However, the specific role of LINC00326 in NSCLC is not known.

MicroRNAs (miRNAs) are stable single-stranded RNAs [21] that negatively modulate gene expression through interaction with the 3′-UTRs of targeted genes [22, 23]. miRNAs have been found to regulate NSCLC pathogenesis [24, 25]. For example, miRNA-21 [26] and miRNA-139-3p [27] involved in the aggressiveness of NSCLC, miRNA-130a was reported to promote the cisplatin resistance and promote the progression of NSCLC via inhibiting PUM-2 expression [28]. Recent evidence has shown cancer-associated links between miRNAs and lncRNAs [29]. LncRNAs may operate as competing endogenous RNAs (ceRNAs) for miRNA binding and regulating mRNA levels [30, 31]. Thus, it is possible that both lncRNAs and miRNAs may be important in NSCLC.

Based on LINC00326 gene expression patterns during spermatogenesis [19], LINC00326 may function in cell proliferation and controlled apoptosis to eliminate irreparably damaged germ cells during development, but the exact regulatory mechanisms remain to be determined. LINC00326 gene expression is almost completely diminished in testicular cancers and gradually decreases with increased testicular cancer severity. This implied that the antiproliferative and proapoptotic properties of LINC00326 peak at the early stages of carcinogenesis [20]. In this study, we hypothesized that LINC00326 is essential in NSCLC progression. The expression of LINC00326 in NSCLC tumor tissues and cell lines was investigated using multiple assays. Through in-depth research on lncRNA and miRNA, it was found that LINC00326 targets the miR-657/DKK2 axis signaling and blocks tumor-related functions in NSCLC cells through the Wnt/β-catenin pathway. This suggests that LINC00326 may be a target for NSCLC therapy (Table 1).

**Table 1:** Sequences for qPCR primers and small interfering RNA (siRNA)

Items	Sequences
Primer for LINC00326	Forward: 5′-GCAGTCCTGTCTCCATTGACT-3′
	Reverse: 5′-ATGGGGTAGGCAGCAAACATA-3′
Primer for miR-657	Forward: 5′-ACACTCCAGCTGGGGGCAGGTTCTCACCC-3′
	Reverse: 5′-CTCAACTGGTGTCGTGGA-3′
	RT: 5′-CTCAACTGGTGTCGTGGAGTCGGCAATTCAGTTGAGCCTAGAGA-3′
Primer for DKK2	Forward: 5′-GAAGGGTTTTGCTGTGCTCG-3′
	Reverse: 5′-ACTTTGCAAGACAGGCCCTT-3′
Primer for U6	Forward: 5′-CTCGCTTCGGCAGCACA-3′
	Reverse: 5′-AACGCTTCACGAATTTGCGT-3′
Primer for GAPDH	Forward: 5′-CTGGGCTACACTGAGCACC-3′
	Reverse: 5′-AAGTGGTCGTTGAGGGCAATG-3′
siRNA for LINC00326	siRNA-395: 5′-UAAUACAACAUAAAUUUACGGdTdT-3′
	siRNA-626: 5′-UAUUCUUCGUGAUGAAAACGAdTdT-3′
	siRNA-1098: 5′-UCUCAAAAAUAAAACUUAGGUdTdT-3′
	siCTRL: 5′-UUCUCCGAACGUGUCACGUdTdT-3′
siRNA for DKK2	siRNA-195: 5′-AGGUUUUUGCCCUUCUUACUGdTdT-3′
	siRNA-386: 5′-UUAAGAUGCUUUCAGUAACUGdTdT-3′
	siRNA-588: 5′-AUUUUGGUCCAGAAAUGACGAdTdT-3′

## Results

### LINC00326 levels are reduced in NSCLC and predict a favorable prognosis

**Fig. 1:**
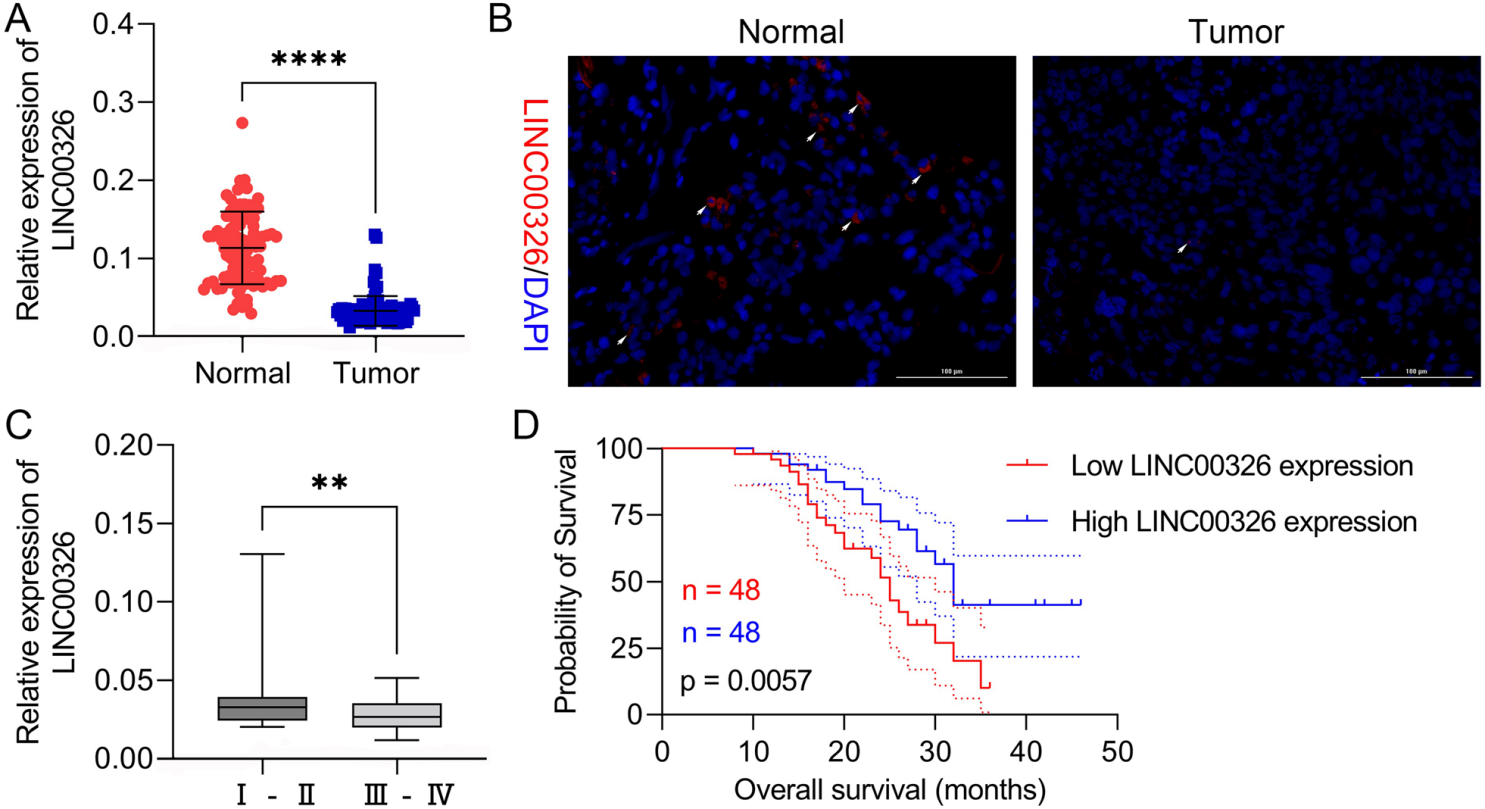
LINC00326 is downregulated and associated with favorable prognosis in NSCLC. **a** Levels of LINC00326 in NSCLC and normal tissues, measured by RT-qPCR and normalized to control values assay (n = 96). **b** LINC00326 levels measured by FISH. **c** LINC00326 levels in relation to NSCLC tumor stage (I&II, III&IV), measured by RT-qPCR. **d** Kaplan–Meier curves showing overall survival as a function of LINC00326 expression. Data are presented as mean ± standard deviation. The experiments were repeated three times. ** P < 0.01; **** P < 0.0001

The LINC00326 levels were examined using RT-qPCR and FISH in 96 paired NSCLC and normal tissues. This showed reduced levels of LINC00326 in NSCLC samples relative to their normal counterparts (Fig. 1a, b). NSCLC patients were divided into two groups according to the median (value = 0.030) expression level of LINC00326. The clinical relevance of this was then evaluated, showing that LINC00326 levels were linked with TNM stage, tumor differentiation, and lymphatic metastasis in NSCLC patients (Table 2). In line with this, we found that LINC00326 was downregulated in stage III and IV NSCLC tissues compared to stages I and II, suggesting that LINC00326 might be involved in NSCLC malignancy (Fig. 1c). After separating the patients into two groups according to LINC00326 levels, Kaplan–Meier curves were applied to evaluate overall survival (OS), finding an association between elevated LINC00326 expression and longer OS (Fig. 1d). These findings suggest the involvement of LINC00326 in NSCLC. Thus, LINC00326 expression is reduced in NSCLC and may represent a good indicator for the prognosis of patients with NSCLC.

**Table 2:** Correlation between LINC00326 expression and clinicopathological features in 96 NSCLC patients

Parameters	Group	N	Expression of LINC00326		P value
			Low, n (%)	High, n (%)	
Age (years)	≤ 58	47	24 (51.06)	23 (48.94)	0.4205
	> 58	49	21 (42.86)	28 (57.14)	
Gender	Female	48	22 (45.83)	26 (54.17)	0.8379
	Male	48	23 (47.92)	25 (52.08)	
Tumor size (cm)	< 3	45	18 (40.00)	27 (60.00)	0.1447
	≥ 3	51	28 (54.90)	23 (45.10)	
TNM stage	I–II	49	18 (36.73)	31 (63.27)	**0.0021**
	III–IV	47	32 (68.09)	15 (31.91)	
Smoking history	Yes	53	24 (45.28)	29 (54.72)	0.7286
	No	43	21 (48.84)	22 (51.16)	
Lymph node metastasis	Yes	50	33 (66.00)	17 (34.00)	**0.0022**
	No	46	16 (34.78)	30 (65.22)	
Differentiation	Well/moderate	49	13(26.53)	36(73.47)	**0.0076**
	Poor	47	25(53.19)	22(46.81)	

*NSCLC* non-small cell lung cancer

### Downregulation of LINC00326 enhances NSCLC cell proliferation and reduces cell apoptosis through Wnt/β-catenin signaling in vitro

**Fig. 2:**
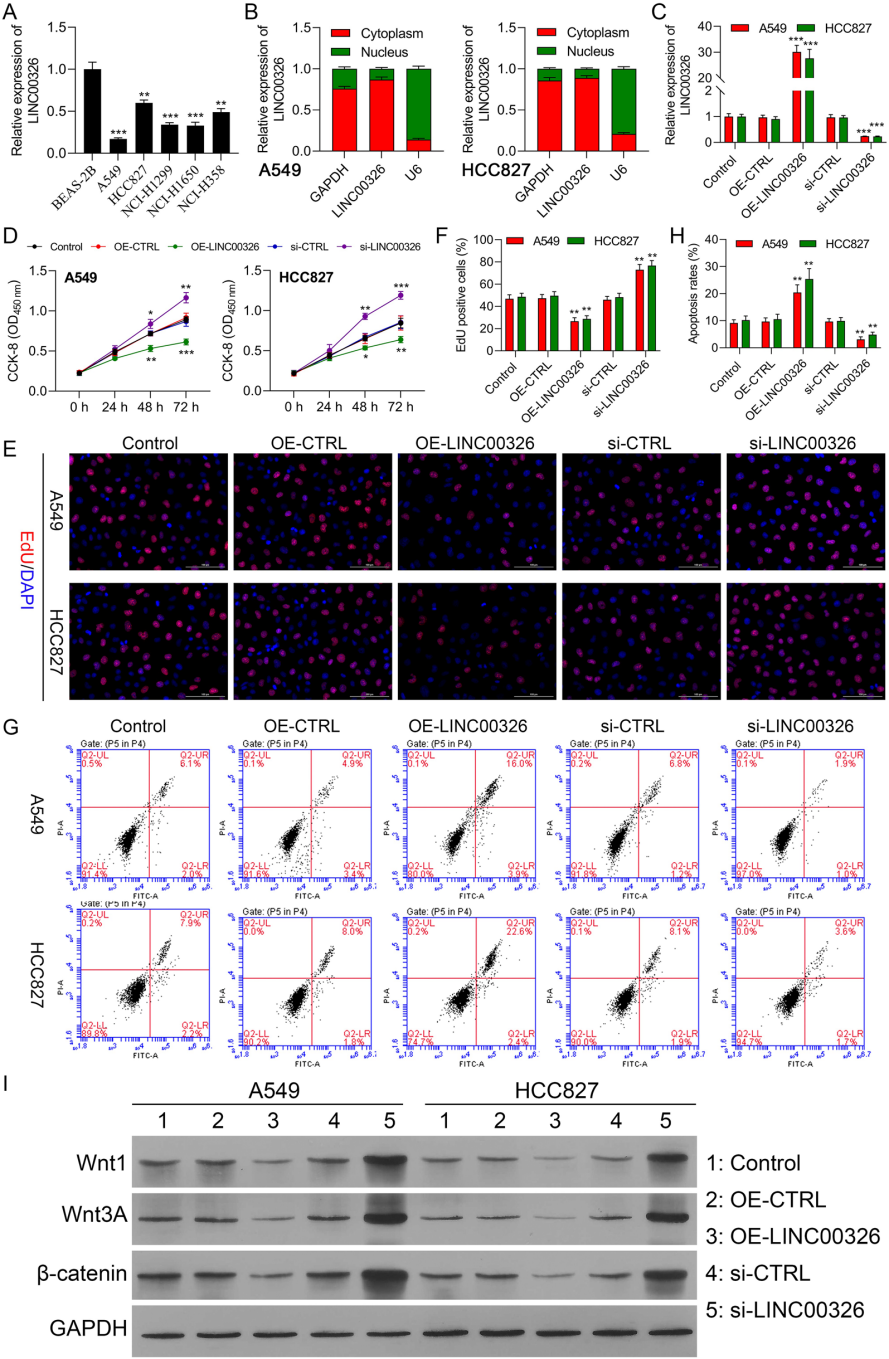
LINC00326 silencing promotes proliferation and reduces apoptosis in NSCLC cells. **a** Relative expression of LINC00326 in NSCLC and normal BEAS-2B cells, measured by RT-qPCR and normalized to controls (equal to 1). **b** Subcellular localization of LINC00326 in A549 and HCC827 cells measured by the isolation of cytoplasmic and nuclear RNA followed by RT-qPCR analysis. **c** Transfection efficiencies of sh-LINC00326 and OE-LINC00326 in A549 and HCC827 cells, measured by RT-qPCR. **d**–**f** Proliferation of transfected A549 and HCC827 cells, measured by CCK-8 and Edu assays. **g**–**h** Apoptosis of transfected A549 and HCC827 cells, measured by flow cytometry. Data are presented as mean ± standard deviation. **i** Wnt1, Wnt3a, and β- catenin protein levels in transfected A549 and HCC827 cells, shown by Western blotting. The experiments were repeated three times. * P < 0.05; ** P < 0.01; *** P < 0.001

Proliferation and apoptosis in A549 and H1650 cells were examined after overexpression or knockdown of LINC00326. LINC00326 levels were analyzed in six cell lines by RT-qPCR. Figure 2a illustrates the LINC00326 levels in the cancer cell lines (A549, HCC827, NCI-H358, NCI-H1299, and NCI-H1650) were markedly lower than those in the non-cancerous BEAS-2B cell line. LncRNAs are documented as ceRNAs to block the miRNA-promoted degradation of specific targets. Thus, the cellular localization of LINC00326 was investigated using RT-qPCR, observing that LINC00326 was mainly localized to the cytoplasm rather than the nucleus in A549 and HCC827 cells (Fig. 2b), indicating that it may function as a ceRNA in NSCLC. Silencing of endogenous LINC00326 was then performed by transfecting A549 cells with si-LINC00326 with siRNA-395 found to be the most efficient in this regard (Additional file 1: Figure S1) was thus used in further investigations. The transfection efficiencies of the overexpressing OE-LINC00326 and silencing si-LINC00326 were examined in A549 and HCC827 cells (Fig. 2c). It was found that LINC00326 silencing increased the viability of both cell lines, while LINC00326 overexpression had the opposite effect (Fig. 2d-f). We then investigated the effects of LINC00326 on apoptosis using flow cytometry, showing elevated apoptosis with LINC00326 overexpression (Fig. 2g-h). Levels of Wnt-related proteins were examined by western blotting. This indicated that LINC00326 overexpression reduced the levels of Wnt1, Wnt3a, and β-catenin, while the proteins were increased after LINC00326 silencing (Fig. 2i and Additional file 1: Figure S2). This shows that LINC00326 reduces NSCLC proliferation and promotes apoptosis in vitro through Wnt/β-catenin activity.

### LINC00326 sponges miR-657

**Fig. 3:**
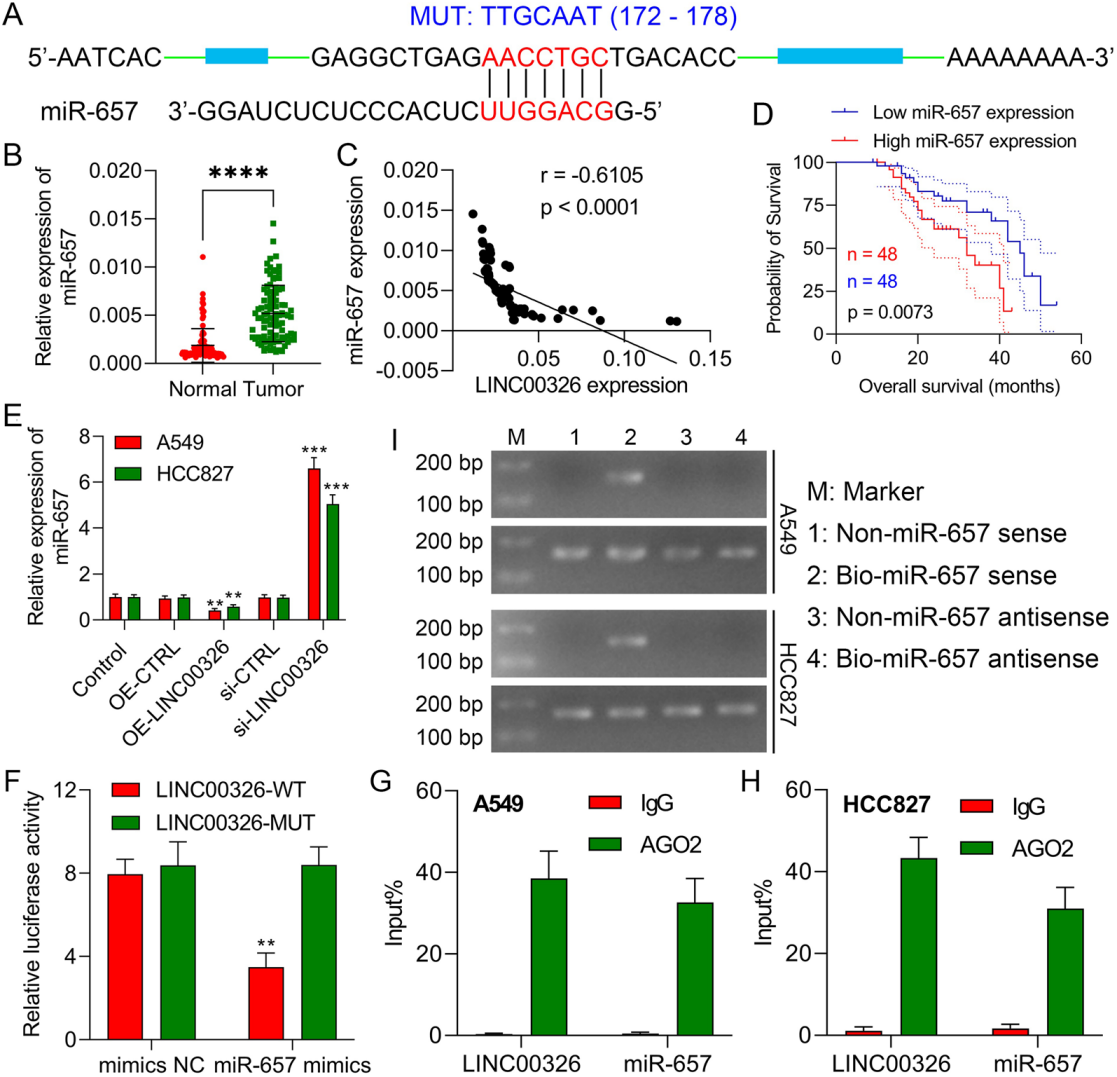
LINC00326 sponges miR-657 in NSCLC cells. **a** Predicted miR-657-binding sequences in LINC00326 (LINC00326-wt) and the design of the mutant sequence (LINC00326-mut). **b** miR-657 mRNA levels in NSCLC and control tissues, measured by RT-qPCR (n = 96); **c** Correlation between LINC00326 and miR-657 levels in tumor samples -n = 96) (r =  − 0. 6105, P < 0.0001). **d** Association between miR-657 levels and patient overall survival. **e** miR-657 mRNA levels in HCC827 and A549 cells transfected with OE-CTRL, OE-LINC00326, si-CTRL, or si-LINC00326, measured by RT-qPCR. **f** Luciferase activity in 293T cells 48 h after transfection with mimics-NC/miR-657 mimic and LINC00326-WT/ LINC00326-MUT. **g**, **h** Interaction between LINC00326 and miR-657 shown by RIP and RT-qPCR. **i** RNA pull-down using biotinylated miR-657. Data are presented as mean ± standard deviation. The experiments were repeated three times. ** P < 0.01; *** P < 0.001. **** P < 0.0001

miRNAs that could bind to LINC00326 were predicted by bioinformatics, including DIANA-LncBase v2 (https://diana.e-ce.uth.gr/lncbasev3), LNCediting (http://bioinfo.life.hust.edu.cn/LNCediting/) and RegRNA 2.0 (http://regrna2.mbc.nctu.edu.tw/). The predicted binding sites for wt and mutant LINC00326 on miR-657 are shown in Fig. 3a. These predictions were then verified in NSCLC and control tissues by investigating miR-657 expression using RT-qPCR. This indicated raised levels of miR-657 in tumor specimens (Fig. 3b). LINC00326 and miR-657 were negatively correlated in NSCLC specimens (Fig. 3c; r =  − 0.6105, P < 0.0001). Then, NSCLC patients were divided into two groups according to the median (value = 0.005) expression level of miR-657. We then examined the relationships between miR-657 and clinicopathological characteristics. As seen in Table 3, miR-657 was significantly linked with clinical stage (P = 0.0022), lymph node metastasis (P = 0.0040), and tumor differentiation (P = 0.0079). However, there were no significant associations between miR-657 and age, sex, tumor size, or smoking history. Consistent with the results in Fig. 3d, Kaplan–Meier curves showed an association between elevated miR-657 levels and reduced OS. Thus, we conclude that miR-657 levels are elevated in NSCLC and related to reduced OS. Next, we examined whether LINC00326 modulated miR-657 expression in NSCLC. The RT-qPCR results showed increased miR-657 expression after LINC00326 silencing and reduced levels after LINC00326 overexpression (Fig. 3e). Furthermore, luciferase activity was lower after co-transfection of the miR-657 mimic and LINC00326-WT, confirming the interaction between miR-657 and LINC00326 (Fig. 3f). Ago2 is critically involved in the RNA-mediated silencing of gene expression, downregulating mRNAs through the action of miRNAs. Thus, we investigated possible direct interactions between LINC00326 and Ago-2 in A549 and HCC827 cell lysates with an anti-Ago-2 antibody in RIP assays. The results illustrated in Fig. 3g-h show significant enrichment of both LINC00326 and miR-657 in Ago2 immunoprecipitates compared with the controls using non-specific IgG, suggesting direct bindings between LINC00326 and miR-657 in NSCLC cells. This finding was then verified using an RNA pull-down assay which showed precipitation of LINC00326 by biotinylated miR-657 (Fig. 3i). Thus, LINC00326 acts as a sponge for miR-657 and is negatively regulated by miR-657 in NSCLC.

**Table 3:** Correlation between miR-657 expression and clinicopathological features in 96 NSCLC patients

Parameters	Group	N	Expression of miR-657		P value
			Low, n (%)	High, n (%)	
Age (years)	≤ 58	47	25 (53.19)	22 (46.81)	0.4164
	> 58	49	22 (44.90)	27 (55.10)	
Gender	Female	48	23 (47.92)	25 (52.08)	0.6820
	Male	48	21 (43.75)	27 (56.25)	
Tumor size (cm)	< 3	45	19 (42.22)	26 (57.78)	0.2941
	≥ 3	51	27 (52.94)	24 (47.06)	
TNM stage	I–II	49	17 (34.69)	32 (65.31)	**0.0022**
	III–IV	47	31 (65.96)	16 (34.04)	
Smoking history	Yes	53	22 (41.51)	31 (58.49)	0.4728
	No	43	21 (48.84)	22 (51.16)	
Lymph node metastasis	Yes	50	31 (62.00)	19 (38.00)	**0.0040**
	No	46	15 (32.61)	31 (67.39)	
Differentiation	Well/moderate	49	14(28.57)	35(71.43)	**0.0079**
	Poor	47	26(55.32)	21(44.68)	

*NSCLC* non-small cell lung cancer

### MiR-657 stimulates proliferation and reduces apoptosis by activating Wnt/β-catenin

**Fig. 4:**
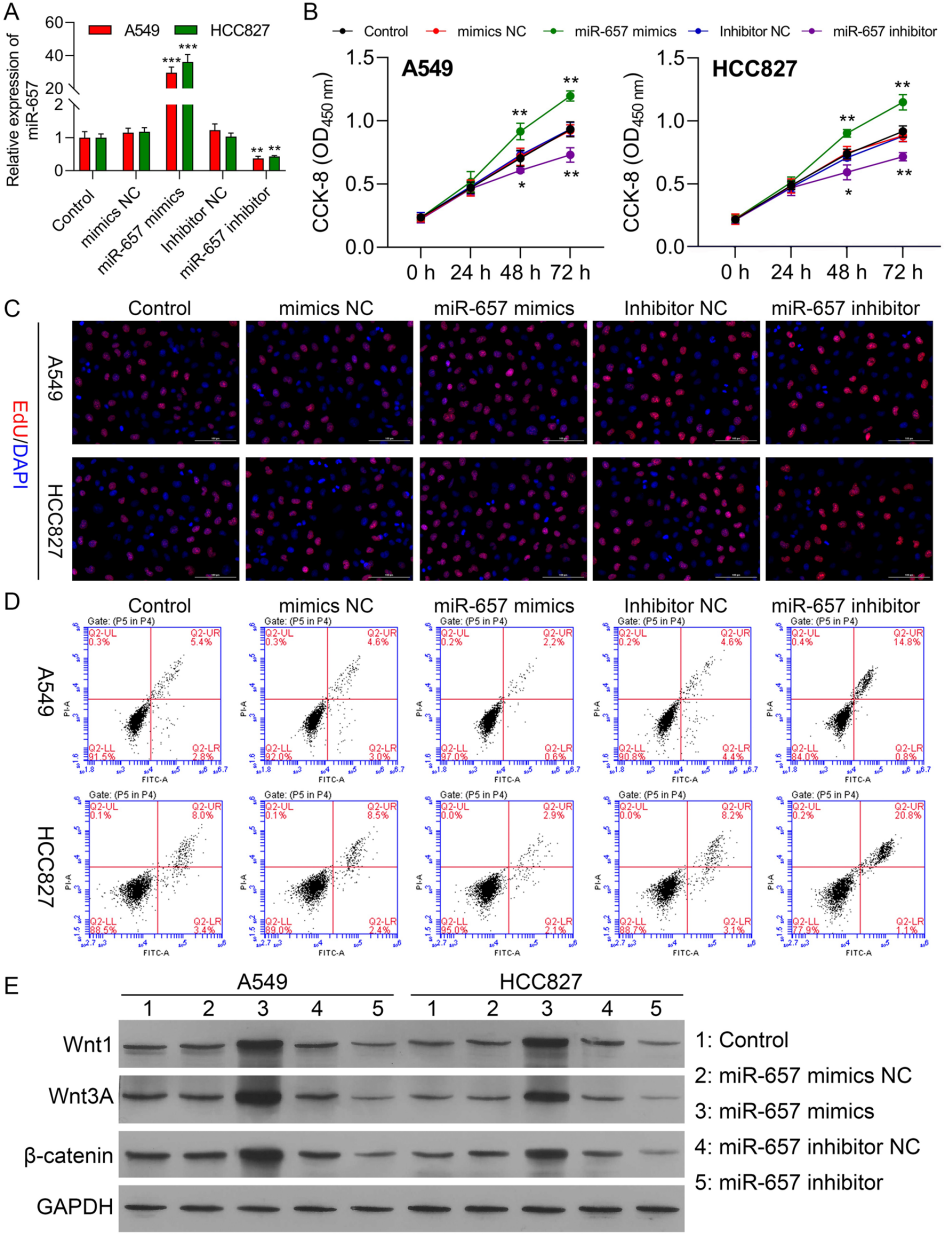
miR-657 stimulates proliferation and reduces promotes apoptosis in A549 and HCC827 cells. **a** Transfection efficiencies of the miR-657 mimic and inhibitor in A549 and HCC827 cells, shown by RT-qPCR. **b, c** Proliferation of transfected A549 and HCC827 cells, measured by CCK-8 and EdU assays. **d** Apoptosis in transfected A549 and HCC827 cells, evaluated by flow cytometry. **e** Levels of Wnt1, Wnt3A, and β-catenin proteins in transfected A549 and HCC827 cells (CTRL, mimic-NC, miR-657 mimics, inhibitor NC, and miR-657 inhibitor groups), shown by western blotting. Data are presented as mean ± standard deviation. The experiments were repeated three times. *P < 0.05; **P < 0.01; ***P < 0.001

To further evaluate the function of miR-657 in NSCLC cells, we transfected cells with miR-657 mimics and inhibitors; the transfection efficiencies are shown in Fig. 4a. CCK-8 results showed that miR-657 overexpression augmented cell viability, while knockdown had the opposite effect (Fig. 4b). EdU assays were used to measure proliferation, showing a marked enhancement of proliferation in miR-657-overexpressing cells compared with the mimics negative controls (mimics NC) (Figs. 4c and Additional file 1: Fig. S3A). Consequently, miR-657 knockdown cells grew slower than the inhibitor negative control group (inhibitor NC). Flow cytometry showed an enhancement of apoptosis after miR-657 knockdown, whereas increased expression of miR-657 upregulation significantly reduced the numbers of apoptotic cells (Fig. 4d and Additional file 1: Fig. S3B). To investigate the hypothesis that miR-657 enhanced proliferation by upregulating the Wnt/β-catenin pathway, we used western blotting to assess Wnt1, Wnt3A, and β-catenin protein levels. Reduced levels of all three proteins were seen after miR-657 downregulation, in contrast to significantly upregulated levels after transfection of the miR-657 mimics **(**Fig. 4e and Additional file 1: Figure S3C). We thus conclude that miR-657 may accelerate NSCLC progression at least partially through the activation of Wnt/β-catenin.

### LINC00326 elevates DKK2 levels in NSCLC cells through competitive binding to miR-657

**Fig. 5:**
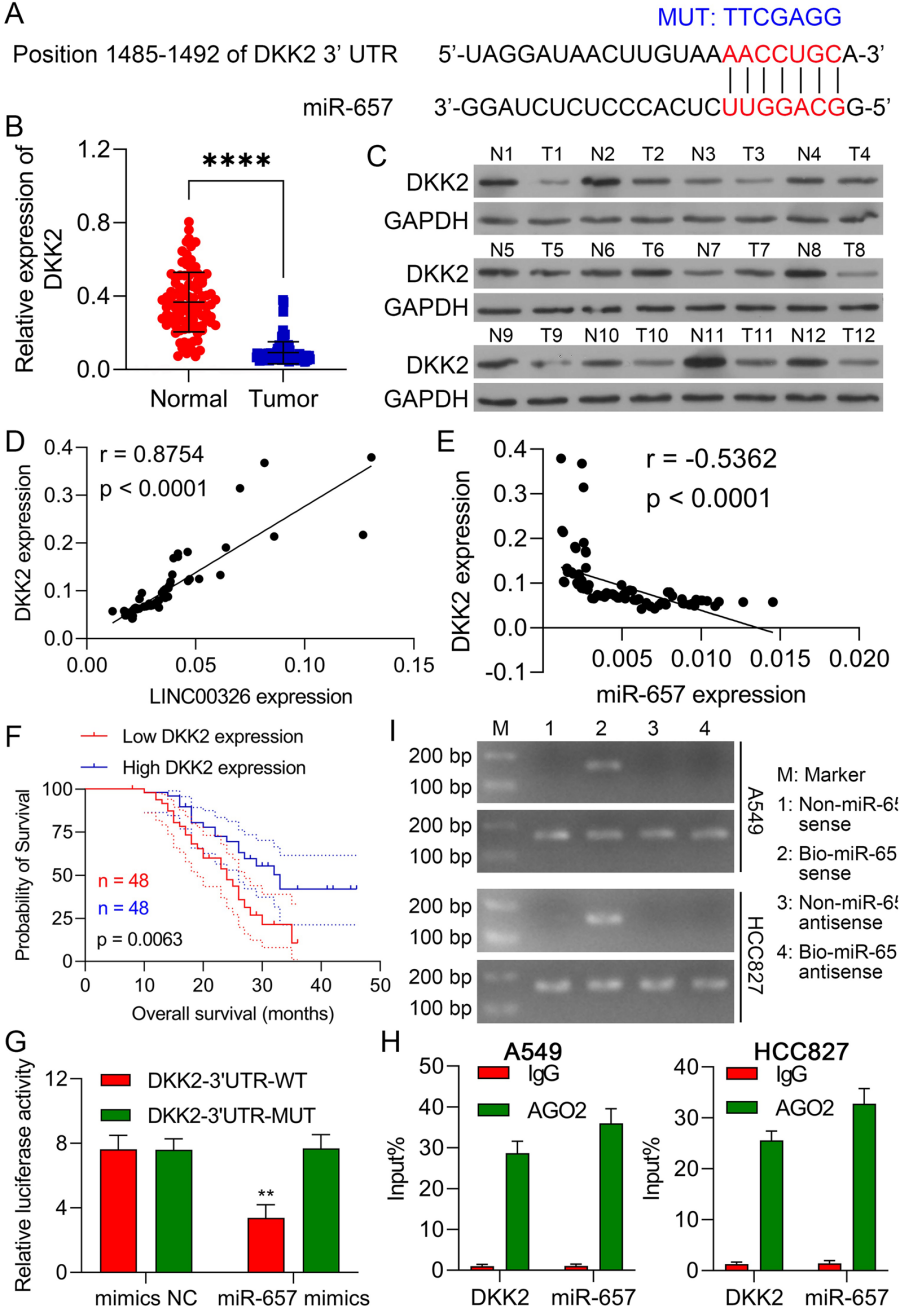
miR-657 targets DKK2 mRNA and DKK2 expression is positively regulated by LINC00326. **a** Schematic view of the miR-657 binding site in the wt and mut 3′-UTR of DKK2 mRNA. **b** DKK2 levels in tumor and normal tissues, measured by RT-qPCR (n = 96). **c** DKK2 protein levels in 12 paired NSCLC and control samples. **d** Correlations between DKK2 mRNA and LINC00326 levels in NSCLC samples (n = 96) (r = 0.8754, P < 0.0001). **e** Correlations between DKK2 mRNA and miR-657 levels in NSCLC tumor samples (n = 96). **f** Kaplan–Meier curves showing relationship between DKK2 levels and overall survival. **g** Relative luciferase activity in 293T cells after co-transfection with the luciferase reporter plasmid (carrying either the wt or mut DKK2 -3′-UTR) and either miR-657 mimics or mimics NC. **h** RIP of DKK2 and miR-657 in A549 and HCC827 cell lysates. Coprecipitated RNA levels were measured by RT-qPCR. **i** RNA pull-down in A549 and HCC827 cells coprecipitated with biotin-labeled hsa-miR-657. Data are presented as mean ± standard deviation. The experiments were repeated three times. **P < 0.01; ***P < 0.001. ****P < 0.0001

Potential miR-657 targets were investigated using the miRBD, TargetScan, and StarBase version 3.0 online databases. This resulted in the prediction of DKK2 as a candidate miR-657 target; the predicted interaction site between miR-657 and the 3′-UTR of DKK2 mRNA is shown in Fig. 5a. The relationships between miR-657 and DKK2 expression in the 96 NSCLC and normal tissue pairs were investigated using RT-qPCR. This indicated downregulation of DKK2 mRNA in the tumor tissues (Fig. 5b), which was confirmed by western blotting (Fig. 5c), together with a negative correlation between DKK2 downregulation and miR-657 levels (Fig. 5e; r =  − 0.5362, P < 0.0001). These findings indicate that miR-657 targets DKK2 mRNA in NSCLC. Then, NSCLC patients were divided into two groups according to the median (value = 0.072) expression level of DKK2. We next examined the clinical significance of DKK2 downregulation in NSCLC. As seen in Table 4, DKK2 levels were closely associated with the TNM stage, tumor differentiation, and lymphatic metastasis in NSCLC patients. The patients were then separated into two groups based on DKK2 expression, and Kaplan–Meier curves were used to analyze DKK2 levels in relation to OS. This indicated that higher DKK2 levels were related to increased OS (Fig. 5f). In addition, DKK2 mRNA and LINC00326 levels were significantly associated with the 96 tumor samples (Fig. 5d; r = 0.8754, P < 0.0001). Furthermore, DKK2 levels were found to be reduced after LINC00326 silencing or transfection of the miR-657 mimics but were increased on LINC00326 overexpression or transfection with miR-657 inhibitors (Additional file 1: Figure S4). Raised levels of miR-657 also significantly reduced DKK2-WT-driven luciferase activity, which was attenuated by mutation of the putative miR-657 interaction site (Fig. 5g). RNA binding protein immunoprecipitation (RIP) experiments showed that miR-657 coprecipitated with DKK2, confirming that miR-657 binds to the 3′UTR of DKK2 (Fig. 5h). RNA pull-down assays also showed a pull-down of DKK2 by biotinylated miR-657 (Fig. 5i). These findings indicate that LINC00326 positively modulates DKK2 levels in NSCLC cells through competitive interaction with miR-657.

**Table 4:** Correlation between DKK2 expression and clinicopathological features in 96 NSCLC patients

Parameters	Group	N	Expression of DKK2		P value
			Low, n (%)	High, n (%)	
Age (years)	≤ 58	47	25 (53.19)	22 (46.81)	0.3110
	> 58	49	21 (42.86)	28 (57.14)	
Gender	Female	48	20 (41.67)	28 (58.33)	0.2203
	Male	48	26 (54.17)	22 (45.83)	
Tumor size (cm)	< 3	45	19 (42.22)	26 (57.78)	0.2941
	≥ 3	51	27 (52.94)	24 (47.06)	
TNM stage	I–II	49	18 (36.73)	31 (63.27)	**0.0042**
	III–IV	47	31 (65.96)	16 (34.04)	
Smoking history	Yes	53	22 (41.51)	31 (58.49)	0.0.2422
	No	43	23 (53.49)	20 (46.51)	
Lymph node metastasis	Yes	50	33 (66.00)	17 (34.00)	**0.0011**
	No	46	15 (32.61)	31 (67.39)	
Differentiation	Well/moderate	49	12 (24.49)	37 (75.51)	**0.0039**
	Poor	47	25 (53.19)	22 (46.81)	

*NSCLC* non-small cell lung cancer

### DKK2 inhibits cell proliferation and promotes apoptosis in NSCLC cells through the Wnt/β-catenin pathway

**Fig. 6:**
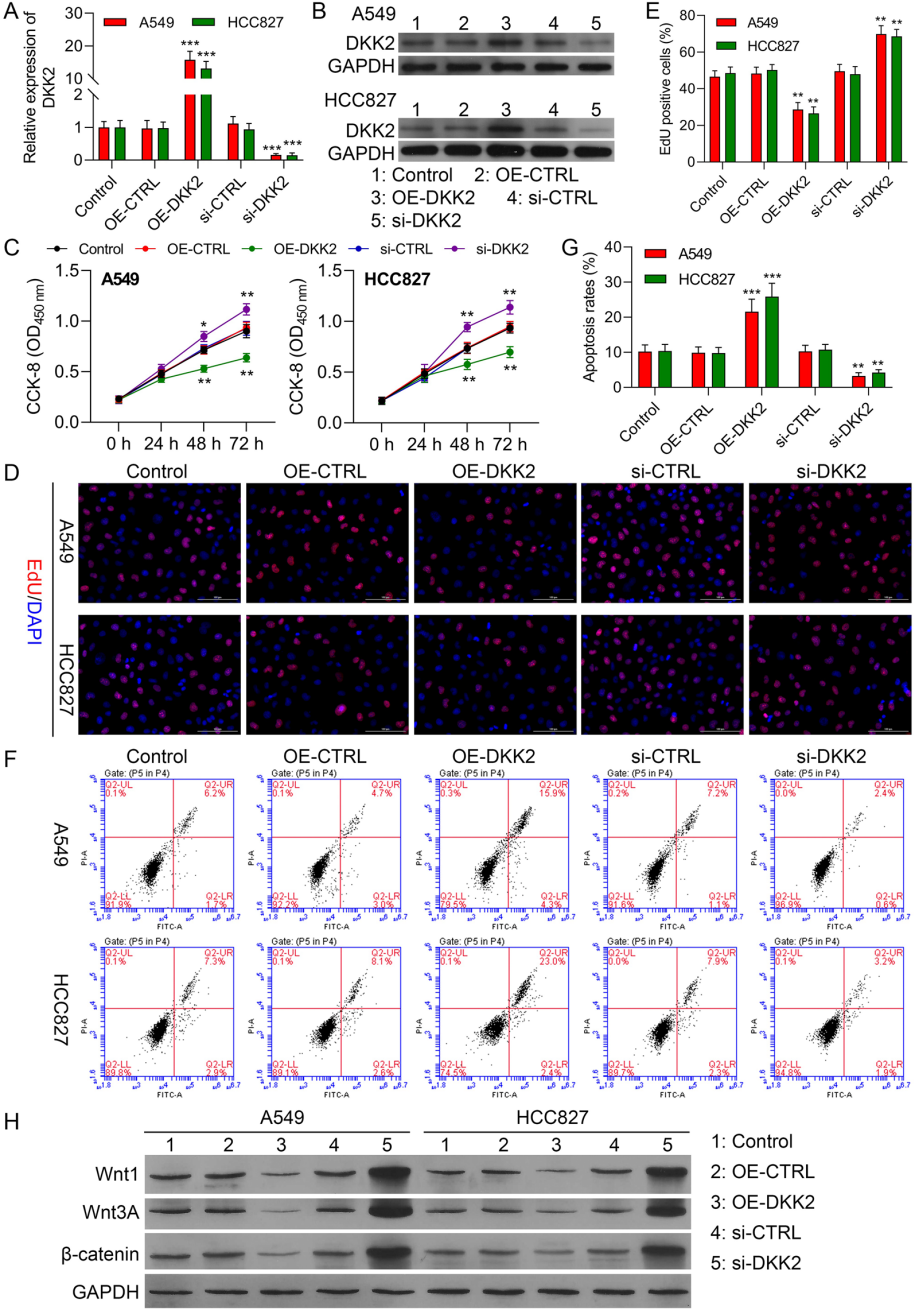
DKK2 reduces proliferation and promotes apoptosis in NSCLC cells. DKK2 was overexpressed or silenced in A549 and HCC827 cells. **a**, **b** DKK2 expression after overexpression or knockdown, shown by RT-qPCR and Western blotting. **c** Proliferation measured by CCK-8 assay. **d**, **e** Proliferation measured by EdU assay. **f**, **g** Apoptosis measured by flow cytometry. **h** Levels of Wnt1, Wnt3A, and β-catenin proteins in transfected A549 and HCC827 cells (Control, OE-CTRL, OE- DKK2, si-CTRL, and si-DKK2), measured by western blotting. Data are presented as mean ± standard deviation. *P < 0.05; **P < 0.01; ***P < 0.001

We transfected A549 cells with siRNA-DKK2 to knock down DKK2 expression. The silencing effect of siRNA-195 was most significant and was therefore used in the subsequent experiments (Additional file 1: Figure S5A-B). Increased DKK2 levels in cells transfected with the overexpression plasmid OE-DKK2 and reduced levels after knockdown was confirmed by both RT-qPCR and Western blotting (Additional file 1: Figures S5C-D and Fig. 6a, b). CCK-8 assays showed that while the proliferation of both A549 and HCC827 cells did not change significantly within 24 h of transfection, proliferation was significantly reduced 48 h after transfection with the OE-DKK2 plasmid (Fig. 6c). In contrast, DKK2 silencing resulted in a significant increase in proliferation (Fig. 6c). This was confirmed by EdU assays (Fig. 6d, e). In addition, flow cytometry showed that DKK2 overexpression enhanced apoptosis while DKK2 silencing reduced apoptosis (Fig. 6f, g). These data demonstrated that DKK2 suppresses NSCLC progression. Next, we used western blotting to assess changes in the expression of Wnt1, Wnt3A, and β-catenin. DKK2 overexpression reduced levels of all three proteins, while DKK2 silencing resulted in marked increases in expression (Fig. 6h and Additional file 1: Figure S6). We thus conclude that DKK2 blocks NSCLC progression by downregulating Wnt/β-catenin signaling.

### Increasing the miR-657/DKK2 axis output counteracts the effects of LINC00326 silencing

**Fig. 7:**
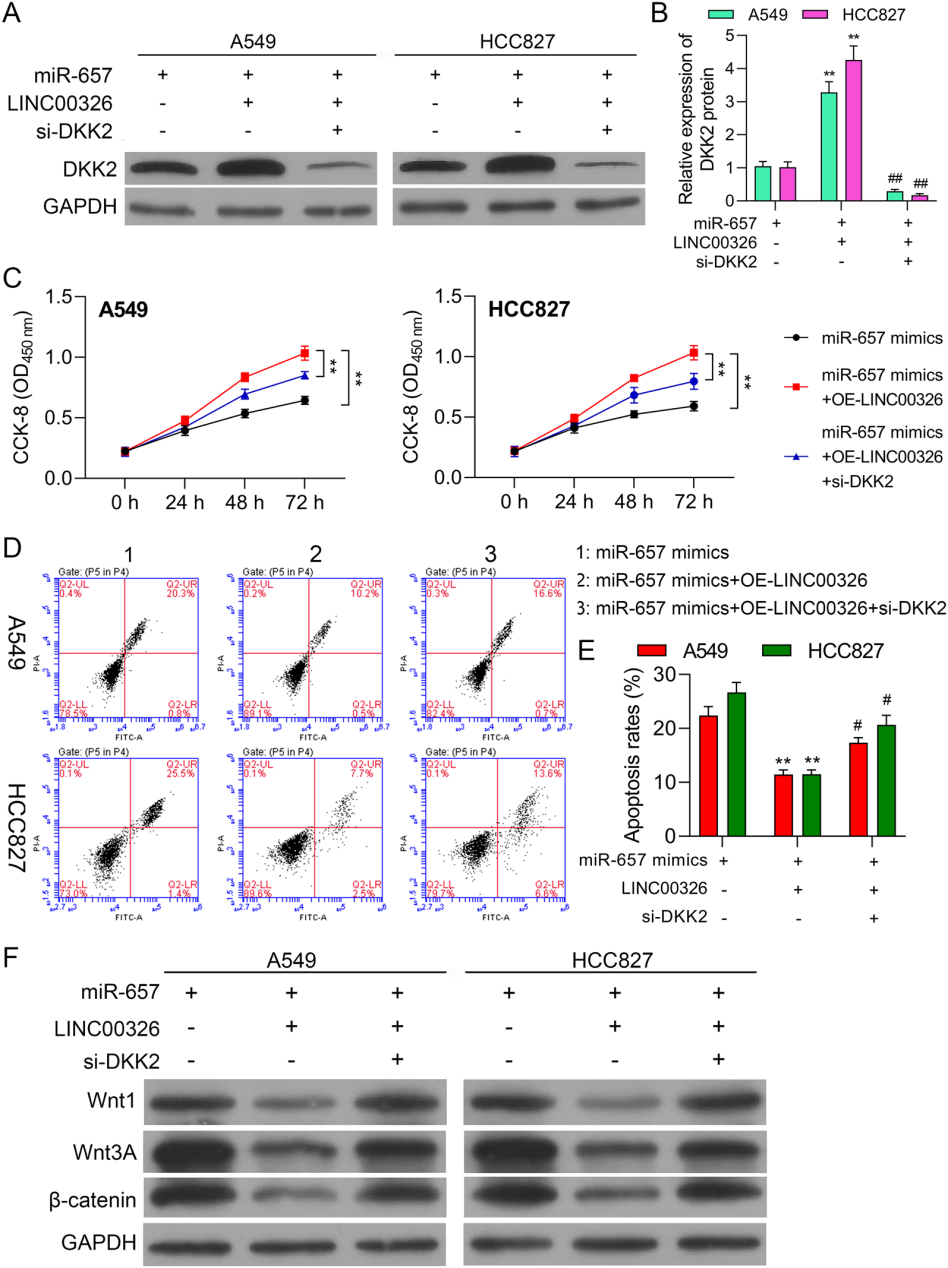
LINC00326 modulates tumor progression and DKK2 expression by interacting with miR-657 in NSCLC cells. A549 and NCI-H1650 cells were transfected with miR-657 mimics and either OE-LINC00326 or OE-LINC00326 along with sh-DKK2. **a** DKK2 protein levels, shown by western blotting. **b** Quantification of different protein expression levels, **P < 0.01 compared with miR-657 mimics transfection group; ##P < 0.01 compared with miR-657 mimics + LINC00326 overexpression plasmids co-transfection group. **c** Cell viability at 24, 48, and 72 h. **d**, **e** Apoptosis measured by flow cytometry, **P < 0.01 compared with miR-657 mimics transfection group; #P < 0.05 compared with miR-657 mimics + LINC00326 overexpression plasmids co-transfection group. **f** Protein levels of Wnt1, Wnt3A, and β-catenin in transfected cells, shown by western blotting with GAPDH as the loading control. Data are presented as mean ± standard deviation. **P < 0.01

We used rescue experiments to assess the influence of miR-657/DKK2 on LINC00326-mediated effects in NSCLC cells. OE-LINC00326 and sh-DKK2 were transfected together with the miR-657 mimics. The DKK2 knockdown was found to reverse the effects of LINC00326 overexpression on DKK2 expression (Fig. 7a, b). Measurement of proliferation by CCK-8 and apoptosis by flow cytometry showed that the increased proliferation and reduced apoptosis induced by miR-657 overexpression were augmented by LINC00326 expression but were partially counteracted by DKK2 knockdown (Fig. 7c–e). In addition, increased LINC00326 expression was found to attenuate the upregulation of Wnt1, Wnt3A, and β-catenin induced by the miR-657 mimic, and these effects were partially abrogated by DKK2 downregulation (Fig. 7f). Thus, these findings suggest that LINC00326 reduces NSCLC progression through the miR-657/ DKK2 axis.

### LINC00326 silencing accelerates tumor growth in vivo

**Fig. 8:**
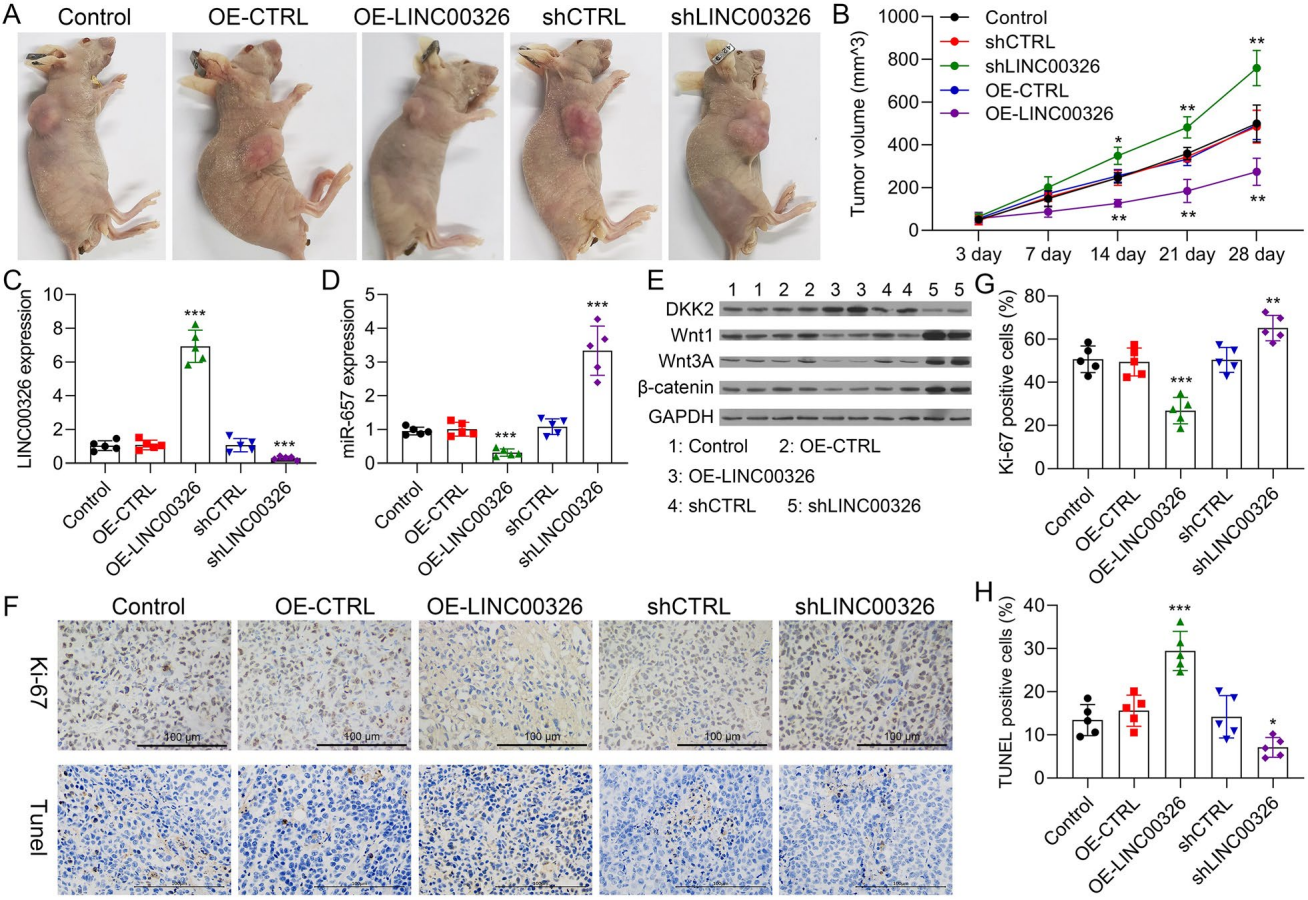
LINC00326 downregulation accelerates tumor growth in vivo. **a** Appearance of tumors four weeks after injection, showing the blank control, negative control, LINC00326 overexpression, shCTRL, and LINC00326 knockdown. **b** Tumor volumes in the five groups (mean ± SD) at time of sacrifice. **c**, **d** LINC00326 and miR-657 mRNA expression in tumors from blank, OE-CTRL, OE- LINC00326, shCTRL, and shLINC00326 groups. **e** Wnt1, Wnt3A, and β-catenin protein levels in tumors from blank, OE-CTRL, OE- LINC00326, shCTRL, and shLINC00326 groups. **f–h** Ki67 and TUNEL staining in blank, OE-CTRL, OE- LINC00326, shCTRL, and shLINC00326 groups, respectively (× 200, scale bars, 100 µm); n = 3. Data are presented as mean ± SEM; *P < 0.05; **P < 0.01; ***P < 0.001

Lastly, the role of LINC00326 in NSCLC tumor growth was investigated in an in vivo xenograft model. A549 cells transfected with the DNA4.0 vector, pcDNA4.0-LINC00326 vector, NC‑sh-RNA, or LINC00326‑shRNA were injected subcutaneously into the left flanks of the athymic nude mice. It was found that, in agreement with the results of the cell experiments, the LINC00326-knockdown tumors were significantly larger than those of control mice, while the LINC00326-overexpressing (OE-LINC00326) tumors were reduced in comparison with the negative control mice (shCTRL), and the tumors of the LINC00326 overexpression group (OE-LINC00326) were significantly smaller than those of the negative control group (OE-CTRL) (Fig. 8a, b). LINC00326 and miR-657 mRNA levels in the different tumors were confirmed by RT-qPCR (Fig. 8c, d) and DKK2 protein levels were examined by western blotting (Fig. 8e and Additional file 1: Figure S7). Immunohistochemistry of the tumor tissues showed that LINC00326 overexpression reduced the levels of the differentiation-associated marker Ki67 (Fig. 8f, g). TUNEL assays confirmed higher levels of apoptosis in the LINC00326-overexpression tumors and lower levels in the LINC00326 knockdown tumors (Fig. 8f, h). Overall, these results indicate that LINC00326 reduces NSCLC growth and differentiation.

The protein levels of Wnt1, Wnt3A, and β-catenin were found to be reduced in the LINC00326-overexpressing xenograft tumors (Fig. 8e), with opposite effects seen in the LINC00326-silenced tumors (Fig. 8e). We thus surmise that LINC00326 reduces NSCLC progression at least partially through activation of Wnt/β-catenin signaling in vivo.

## Discussion

The tumorigenic roles of lncRNAs have been documented in a variety of human cancers [32–34], including NSCLC [35–37]. This indicates that lncRNAs may be significant targets for diagnosing, treating, and predicting the prognosis of NSCLC. Although the association of a number of lncRNAs with NSCLC has been verified [31], further investigation into their precise roles is needed. This study focuses on the role of LINC00326 in the progression of NSCLC in vitro and in vivo. Our study found that LINC00326/miR-657/DKK2 axis signaling blocks tumor-associated functions in NSCLC cells by targeting the Wnt/β-catenin pathway. This suggests that LINC00326 may be a target for NSCLC therapy.

Recently, Sondergaard et al. [38] showed that LINC00326 modulates lipid metabolism by interacting with RBP CCT3. It has also been found to influence spermatogenesis and is differentially expressed in infertility [19]. In our previous sequencing results, we found that LINC00326 was downregulated in NSCLC tissues compared with normal tissues (n = 6, data not shown). However, its role in NSCLC has not been determined. Here, LINC00326 levels were measured by RT-qPCR was performed on 96 pairs of human NSCLC tumors and control tissues with significantly lower levels observed in the tumor. Five NSCLC cell lines were also used; these also showed lower LINC00326 levels than normal BEAS-2B cells. Functionally, the interfKnockdown of LINC00326 both elevated proliferation and reduced apoptosis in NSCLC cells in vitro, while promoting tumor growth in xenograft models in *vivo*. We then investigated the mechanisms by which LINC00326 exerts its anti-oncogenic effects. It has been found that lncRNA function is associated with their subcellular localization [39, 40]. We thus investigated the localization of LINC00326 in A549 and HCC827 cells using cell fractionation and RT-qPCR, observing that LINC00326 was mainly localized to the cytoplasm. The ceRNA hypothesis states that lncRNAs function as sponges for miRNAs in cancer, leading to reduced degradation or suppression of mRNA targets [41, 42]. This crosstalk between lncRNAs and miRNAs modulates gene expression and may thus regulate cancer development and progression [29, 43]. Here, miR-657 was predicted to be a target for LINC00326. This was confirmed using luciferase reporter assays showing a direct interaction between LINC00326 and miR-657, as well as by RIP assays. Furthermore, miR-657 also showed elevated expression in tumor tissues. Direct interaction between miR-657 and the DKK2 3′-UTR was also observed. Interestingly, LINC00326 knockdown raised miR-657 levels while reducing those of DKK2 through miR-657 sponging. LINC00326 levels were negatively associated with those of miR-657 but positively associated with the DKK2 levels in the 96 NSCLC tissues. Thus, these findings demonstrated a novel LINC00236-associated ceRNA network connecting LINC00326, miR-657, and DKK2 in NSCLC.

Prvious studies have identified that miR-657 was closely associated with transcriptional misregulation in aldosterone synthesis and secretion [44]. Aberrant expression of miR-657 has also been found in several human cancers, including hepatocellular carcinoma (HCC) and retinoblastoma [45, 46]. Zhang et al. [46] showed that miR-657 targets transducin-like enhancer protein 1 by NF-κB activation in HCC, promoting tumorigenesis. Jin et al. [47] demonstrated that miR-657 may play critical regulatory roles in lung tumor development. Moreover, the levels of miR-657 have also been found to predict the recurrence of resected stage I NSCLC tumors [48]. Here, we observed that LINC00326/miR-657 modulated the expression of DKK2. The rescue experiments also showed that the effects of LINC00326 silencing were mitigated by stimulation of the miR-657 /DKK2 axis, suggesting that miR-657/DKK2 was necessary for the LINC0326 promotion of NSCLC progression.

Dickkopf 2 (DKK2) is a member of the Dickkopf family and was identified as a modulator in the Wnt / β-catenin pathway via binding of the lipoprotein receptor-related protein 5/6 [49]. The Wnt pathway is closely associated with development and stemness and has also been linked with cancer [50]. Although the latter has been extensively described in colorectal cancer, abnormal Wnt signaling has been noted in numerous other tumor types [51]. Its hyperactivation can lead to aberrant cell growth and tumor progression. Previous studies showed that DKK2 expression is modulated by expression of microRNA-221 (miRNA-221) in esophageal cancer [52]. Fang et al. [53] demonstrated that overexpression of miR-582-3p maintains stemness through negative modulation of the Wnt regulators Axin2, DKK3, and SRP1, leading to an increase in β-catenin-promoted Wnt activity in NSCLC. It is known that DKK2 antagonizes Wnt signaling [54], thus, inactivation of DKK2 increases Wnt activity with accelerating tumor progression, and activation of the Wnt/β-catenin pathway has been documented in NSCLC [55]. It is thus possible that miR-657 promotes tumorigenesis through DKK2-mediated modulation of Wnt/ β-catenin signaling. We thus investigated the levels of β-catenin and downstream Wnt/β-catenin genes. It was found that miR-657 overexpression reduced DKK2 expression, thus abrogating the inhibition of Wnt/β-catenin signaling by DKK2. Activated β-catenin translocates to the nucleus, activating downstream effectors and contributing to tumorigenesis [56]. Here, we showed for the first time that miR-657 acts as an onco-miR in NSCLC by targeting DKK2 and activating the Wnt/β-catenin signaling.

For the limitation, in the present study, only one siRNA was used to knockdown the target gene to investigate the role of LINC00326 and DKK2 on NSCLC proliferation and apoptosis. To establish the stable knock-out cancer cell line, two or more siRNAs and even crisper cas-9 technique need to conduct the results more reliably.

## Conclusions

**Fig. 9:**
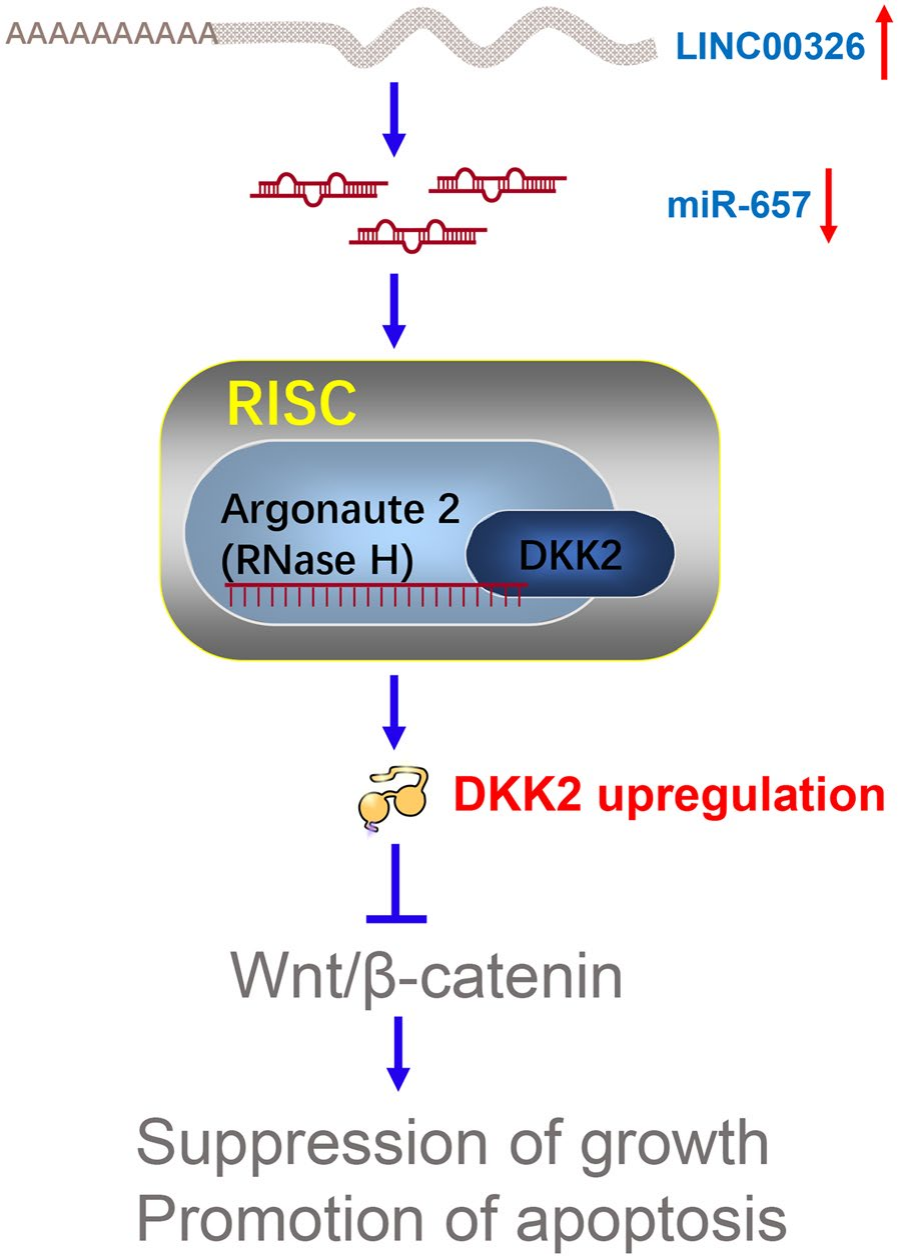
Schematic overview of the mechanistic basis for the observed study results. LINC00326 targets the miR-657/DKK2 pathway to control NSCLC development, while miR-657 acts through the targeting of DKK2, and that disruption of this process leads to tumor development mediated by Wnt/β-catenin signaling

These findings thus suggest that LINC00326 targets the miR-657/DKK2 pathway to control NSCLC development, while miR-657 acts through the targeting of DKK2, and that disruption of this process leads to tumor development mediated by Wnt/β-catenin signaling (Fig. 9).

## Methods

### Tissues and cells

Ninety-six tumor samples and their adjacent normal tissues (2 cm from the lesion) were obtained from patients with primary NSCLC in the Sichuan Cancer Hospital & Institute, Sichuan Cancer Center, between March 2014 and September 2016. None of the patients had received radiotherapy or chemotherapy before the surgical operation. The study was approved by the Ethics Committee of Sichuan Cancer Hospital & Institute, Sichuan Cancer Center and was conducted following the Declaration of Helsinki (2013 revision). The patients provided informed written consent. Samples were kept in liquid nitrogen for analysis.

The human NSCLC cell lines A549, NCI-H1299, HCC827, NCI-1650, and NCI-H358 were acquired from the National Collection of Authenticated Cell Cultures (Shanghai, China), and the normal human lung epithelial cell line DEAS-2B was obtained from Beyotime (Shanghai, China). A549 cells were maintained in DMEM/F12 medium (Procell, Wuhan, China) with 10% fetal bovine serum (FBS; Gibco, Waltham, MA, USA). The remaining cell lines were cultured in RPMI 1640 medium (Procell, Wuhan, China) containing 1% Glutamax (Invitrogen), 1% (v/v) 100 mM sodium pyruvate solution (Invitrogen, Waltham, MA, USA), and 10% FBS. All cells were grown in a humidified 37 °C incubator with 5% CO_2_.

### Cell transfections

LINC00326 and DKK2 small interfering (si) RNA (siLINC00326, and siDKK2), scrambled siRNA (si-negative control (57)) (Table 1) and a pcDNA3.1-LINC00326 overexpression plasmid were designed and synthesized by Shanghai GenePharma Co., Ltd. (Shanghai, China). miR-657 mimics, NC miRNA mimic (mimic NC), miR-657 inhibitor, and inhibitor NC were acquired from Guangzhou RiBoBio Co., Ltd. (Guangzhou, China). The pcDNA 4.1 plasmid specific to DKK2 (termed OE-DKK2) and empty pcDNA4.1 plasmid were purchased from GeneChem Company (Shanghai, China). Cells were plated in 6-well plates and transfected using Lipofectamine 2000 (Invitrogen).

### Cell fractionation

Cytoplasmic and nuclear fractions were separated with a PARIS kit (Invitrogen). Total RNA was extracted from both fractions and the concentrations of LINC00326, GAPDH (cytoplasmic loading control), and U6 small nuclear RNA (nuclear loading control) were determined by RT-qPCR.

### Fluorescence in Situ Hybridization (FISH)

FISH was conducted using a RiboBio FISH kit (Guangzhou, China). Cells were rinsed in PBS and fixed in 4% formaldehyde for 10 min. After permeabilization in PBS with 0.5% Triton X100 at 4 °C for 5 min and three 5-min washes in PBS, the cells were prehybridized at 37 °C for 30 min. Hybridization with an anti-LINC00326 oligodeoxynucleotide probe was performed overnight at 37 °C in the dark. The cells were then counterstained with DAPI and imaged under a confocal laser-scanning microscope (Carl Zeiss, Germany).

### Real-time fluorescence quantitative polymerase chain reaction (RT-qPCR)

TRIzol reagent (Invitrogen) was used for the extraction of total RNA. RNA concentrations and quality were assessed with a NanoDrop 2000 (Thermo Fisher) and the RNA was reverse-transcribed to cDNA using a PrimeScript RT Reagent Kit (Takara, Dalian, China). SYBR Premix Ex Taq (Takara) was used for measuring LINC00326 and DKK2 expression with GADPH as the internal control. For miR-657, total RNA was isolated with the miRNeasy Mini Kit (Qiagen GmbH, Hilden, Germany) and cDNA was synthesized with a miScript Reverse Transcription kit (Qiagen GmbH). The cDNA was assessed by qPCR using the miScript SYBR Green PCR kit (Qiagen GmbH) and expression was normalized to U6. All reactions were performed on an Applied Biosystems 7500 Fast Real-Time PCR system (Applied Biosystems, Waltham, MA, USA), and relative gene expression was determined by the 2^−ΔΔCt^ method. Primers are compiled in Table 1.

### Cell counting kit-8 (CCK-8) assay

Following transfection, cells were grown for 24 h before harvesting and suspension in complete culture media. The cells (2 × 10^3^/well) were seeded in 96-well plates and grown under normal conditions for 1, 24, 48, and 72 h at which point 10 μL of CCK-8 solution (Sigma-Aldrich, St Louis, MO, USA) were added and the cells incubated for a further 2 h. Absorbances at 450 nm were measured using a Tecan microplate reader (Tecan Group, Ltd.).

### EdU staining

EdU staining was performed using the EdU Cell Proliferation Kit (C0075S, Beyotime) according to the product specification. Briefly, 1 × 10^5^ treated A549 and HCC827 cells were cultured into a 6-well plate for 24 h, then A549 and HCC827 cells were incubated with 15 μM EdU solution at 37 ℃ for 2 h, and then fixed with paraformaldehyde and redyed with 2-(4-Amidinophenyl)-6-indolecarbamidine dihydrochloride (DAPI) solution (C1005, Beyotime, Shanghai, China) for 10 min at room temperature, and finally observed with fluorescence microscope (Olympus, Japan).

### Flow cytometry

Transfected cells were incubated for 48 h. Apoptosis was investigated before harvesting and plating in 6-well plates an Annexin V–fluorescein isothiocyanate (FITC) apoptosis detection kit (BioLegend, San Diego, CA, USA). After resuspension in 100 μL of 1 × binding buffer, 5 μL each of Annexin V–FITC and propidium iodide (PI) were added and the cells were incubated in the dark at room temperature for 15 min. The proportions of apoptotic cells were analyzed using a FACSCalibur Flow Cytometry system (BD Biosciences, San Jose, CA, USA) and CellQuest version 2.9 (BD Biosciences).

### Bioinformatics analysis

Interactions were predicted with StarBase version 3.0 (http://starbase.sysu.edu.cn/) and miRDB (http://mirdb.org/), and TargetScan (http://www.targetscan.org/) databases were used for the prediction of miR-657 targets. And DIANA-LncBase v2 (https://diana.e-ce.uth.gr/lncbasev3), LNCediting (http://bioinfo.life.hust.edu.cn/LNCediting/) and RegRNA 2.0 (http://regrna2.mbc.nctu.edu.tw/) were used for predicting the downstream miRNAs of LINC0032.

### Luciferase reporter assay

Sequences containing the predicted wild-type (wt) and mutated (mut) LINC00326 miR-657 binding site were inserted into a pmirGLO Dual-Luciferase vector (Promega, Madison, WI, USA) to form the LINC00326-wt and LINC00623-mut plasmids, respectively. Similarly, DKK2-wt and DKK2-mut reporter plasmids were constructed. 293 T cells were plated in 24-well plates and transfected with miR-657 or control (NC) mimics and either the wt or mut reporter plasmids using Lipofectamine 2000 and grown for 48 h when the firefly and Renilla (for normalization) luciferase activities were determined using the Dual-Luciferase Reporter Assay System (Promega).

### RNA Immunoprecipitation (RIP) Assay

Immunoprecipitation was performed with a Magna RIP RNA-Binding Protein Immunoprecipitation Kit (Millipore, Billerica, MA, USA), according to the product manual. The Argonaute-2 (AGO-2) antibody (Abcam, ab186733) was used for RIP. 1 × 10^6^ of cells were lysed in RIPA buffer and treated with magnetic beads coated with anti-Ago2 or normal IgG before incubating overnight at 4 °C. The beads were washed with chilled NT2 buffer and incubated with proteinase K. The co-immunoprecipitated RNAs were used to detect LINC00326 and miR-657 enrichment by RT-qPCR.

### RNA pull-down

Biotin-conjugated miR-657 (50 nM), Biotin-conjugated miR-657 antisense (50 nM), miR-657 mimics (50 nM), miR-657 antisense (50 nM) were transfected into A549 and HCC827 cells using Lipofectamine™ 2000 reagent (Invitrogen) and cultured for 48 h. After that, cells were harvested (1 × 10^7^ cells) and the cells were lysed, then500 μl of the lysates were incubated with 500 μl of streptavidin-conjugated magnetic beads (Life Technologies) at room temperature for 2 h. After washing, the RNA was extracted with TRIzol and evaluated by PCR.

### Western blotting

Cells were lysed in RIPA buffer containing 1% PMSF and 1% protease inhibitor cocktail (KeyGEN BioTECH; Nanjing, China). Total protein was measured with a BCA Protein Assay Kit (KeyGEN BioTECH) and equal amounts of protein (25 μg) were separated on 10% SDS-PAGE followed by electroblotting to polyvinylidene fluoride (PVDF) membranes. After blocking with 5% fat-free milk at room temperature for 2 h, the membranes were incubated with 1:1000 dilutions of primary antibodies against DKK2 (cat. No. ab38594; Abcam), Wnt1 (cat. No. ab15251; Abcam), Wnt3A (cat. No. ab219412; Abcam), β-catenin (cat. No. ab32572; Abcam), or GAPDH (cat. No. ab8245; Abcam) overnight at 4 °C overnight. The membranes were washed and incubated with the secondary HRP-conjugated goat anti-rabbit IgG (1:5000 dilution; cat. No. ab205719; Abcam) and visualized by enhanced chemiluminescence (GE Healthcare Life Sciences, Little Chalfont, UK). Representative Western blot images were from three independent repeated experiments.

### Tumor xenograft models

All animal studies followed the guidelines of the Sichuan Cancer Hospital & Institute, Sichuan Cancer Center. Nude BALB/c mice (4 weeks old, male) were obtained from GemPharmatech Co. Ltd. (Nanjing, China). The mice were housed under pathogen-free conditions and were randomly assigned to five groups of five mice each. A549 cells (0.5 × 10^7^; WT, and stably transfected with the pcDNA4.0 vector, pcDNA4.0-LINC00326 vector, NC‑shRNA [5′- AATTCAAAAAATTCTCCGAACGTGTCACGTTCTCTTGAATCAGAAAGTTGCTCTTCAGCCG-3′], and LINC00326‑shRNA[5′-AATTCAAAAAATAATACAACATAAATTTACGGTCTCTTGAAGTAGCACAACATTCTCCACCCG-3′]) were injected subcutaneously into the left flanks. Tumor volumes were evaluated weekly for four weeks, measured with calipers and determined as V = length × width^2^ × 0.5. The mice were sacrificed after four weeks and the tumors were extracted and fixed in 4% paraformaldehyde for 24 h. Tumor tissues were stained for TUNEL analysis and immunohistochemically for ki67 using an anti-ki67 antibody (CST; #9449; 1:400). Stained cells were imaged under a brightfield microscope (Olympus, Tokyo, Japan). All animal experiments were done in the animal laboratory center per the study protocol according to the NIH Guide for the Care and Use of Laboratory Animals, approved by the Animal Care and Use Committee of Sichuan Cancer Hospital & Institute, Sichuan Cancer Center.

### Immunohistochemical (IHC) analysis

The expression levels of Ki-67 protein in xenograft tissues were detected by immunohistochemistry (IHC). Anti-Ki-67 antibody (BOSTER, A00254) was used for IHC, as per a previously reported method [58]. IHC staining was captured under microscopy, and the number of Ki-67-positive cells among the total number of cells was counted.

### Terminal deoxynucleotidyl transferase dUTP nick end labeling (TUNEL) assay

TUNEL assay was performed using the Colorimetric TUNEL Apoptosis Assay Kit (Beyotime, Shanghai, China). Briefly, 3 μm of tissue sections from xenograft were dewaxed and incubated with 20 μg/mL protease K without DNase at room temperature for 15 min. After washing with PBS three times, the sections were blocked with blocking buffer (P0100B, Beyotime, Shanghai, China) at room temperature for 20 min, then washed the sections with PBS three times and incubated with 50 μL of biotin labeling solution at room temperature for 1 h in the dark. Subsequently, stained sections were incubated with termination reaction fluid for 10 min at room temperature and incubated with 50 μL of streptavidin-HRP working solution. Finally, sections were incubated with horseradish peroxidase (HRP)-conjugated antibody and the number of TUNEL-positive cells among the total number of cells was counted.

### Statistical analysis

Data were analyzed with SPSS version 18.0 (SPSS, Inc.). Differences between two groups were assessed by t-tests and those between multiple groups by one-way ANOVA and Dunnett’s post hoc test. Correlations between LINC00326 and miR-657 were measured using Pearson’s correlation coefficients. Results were presented as mean ± SD, and P-values < 0.05 were considered statistically significant.

## Supplementary Information

**Additional file 1**. Supplementary figures.

## Data Availability

The datasets used and/or analysed during the current study are available from the corresponding author on reasonable request.

## Abbreviations

LINC00326: Long intergenic non-coding RNA 326
NSCLC: Non-small cell lung carcinoma
lncRNAs: Long non-coding RNAs
FISH: Fluorescence in situ hybridization
ceRNAs: Competing endogenous RNAs
miRNAs: MicroRNAs
CCT3: Chaperonin containing TCP1 subunit 3
GNB5: G protein subunit beta 5
DKK2: Dickkopf WNT signaling pathway inhibitor 2
siRNA: Small interfering RNA
NC: Negative control
PI: Propidium iodide
FITC: Fluorescein Isothiocyanate
WT: Wild-type
MUT: Mutated
CCK-8: Cell Counting Kit-8
IHC: Immunohistochemistry
TUNEL: Terminal deoxynucleotidyl transferase dUTP nick end labeling
SD: Standard deviation
