# Supplementary material for: Upregulation of long intergenic non-coding RNA LINC00326 inhibits non-small cell lung carcinoma progression by blocking Wnt/β-catenin pathway through modulating the miR-657/dickkopf WNT signaling pathway inhibitor 2 axis
Source: Biol Direct. 2023 Feb 6;18:3. doi: 10.1186/s13062-023-00359-9 (PMC9901116; doi:10.1186/s13062-023-00359-9)
Supplement: Supplementary file 1 — Additional file 1. Supplementary figures. [file 13062_2023_359_MOESM1_ESM.docx]

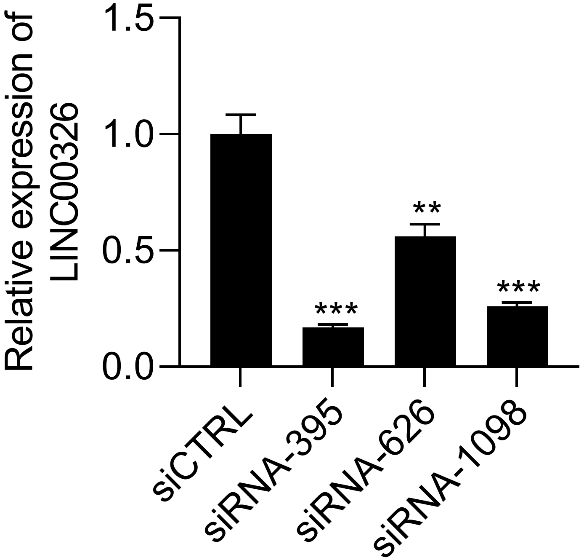


**Figure S1.** A549 cells transfected with si-LINC00326 or si-NC and RT-qPCR determination of the knockdown efficiency. **P < 0.01; ***P < 0.001.


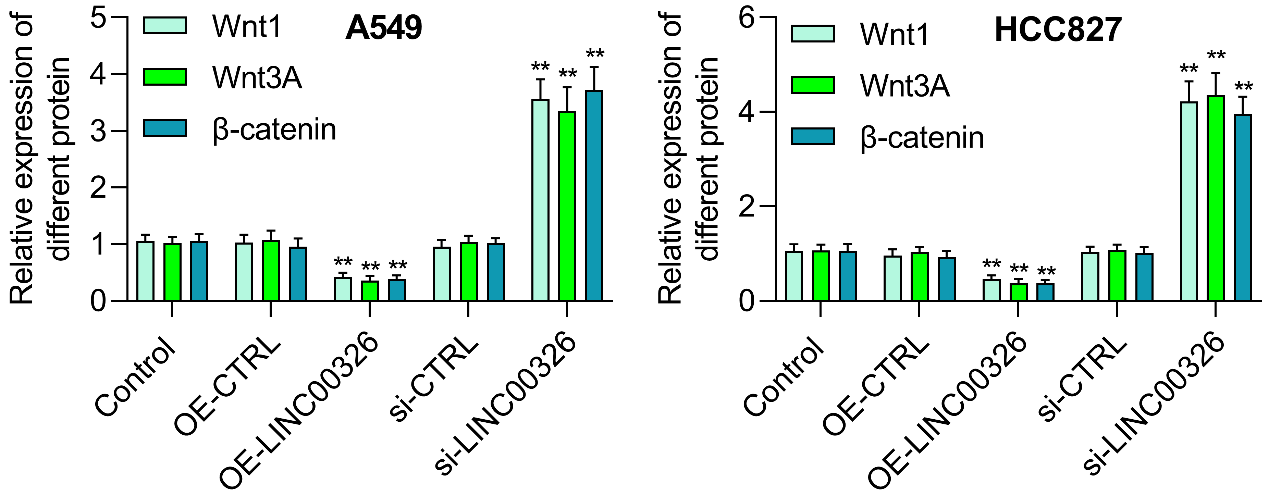


**Figure S2.** **Quantification of different protein expression levels.** Overexpression of LINC00326 suppressed Wnt1, Wnt3A and β-catenin protein expression, while knockdown of LINC00326 induced Wnt1, Wnt3A and β-catenin protein expression. **P < 0.01.


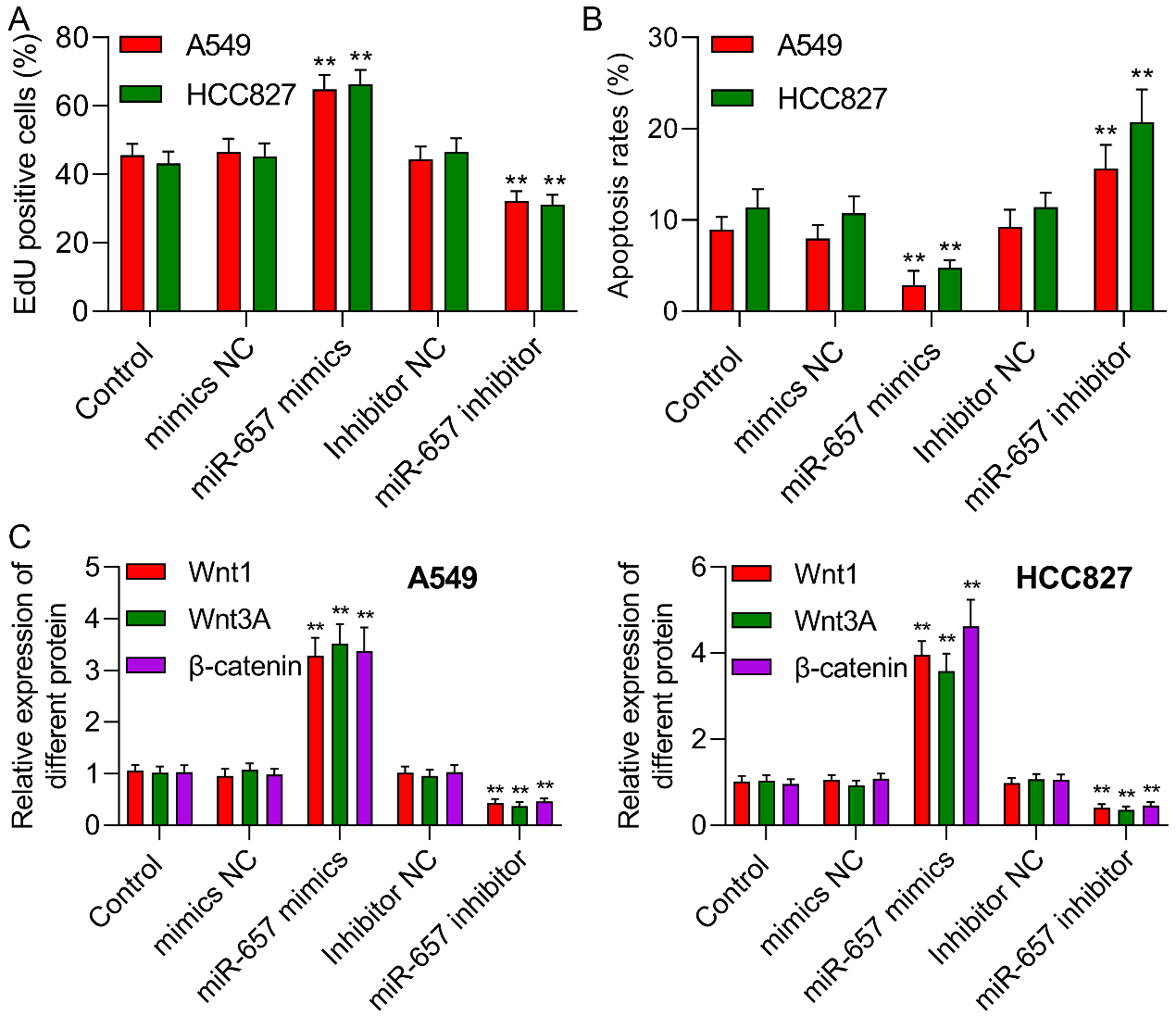


**Figure S3.** Transfection of A549 and HCC827 cells with the miR-657 mimic or inhibitor proliferation measured by EdU assay **(A)** and apoptosis measured by flow cytometry **(B)**. Quantification of different protein expression levels **(C)**. **P < 0.01.


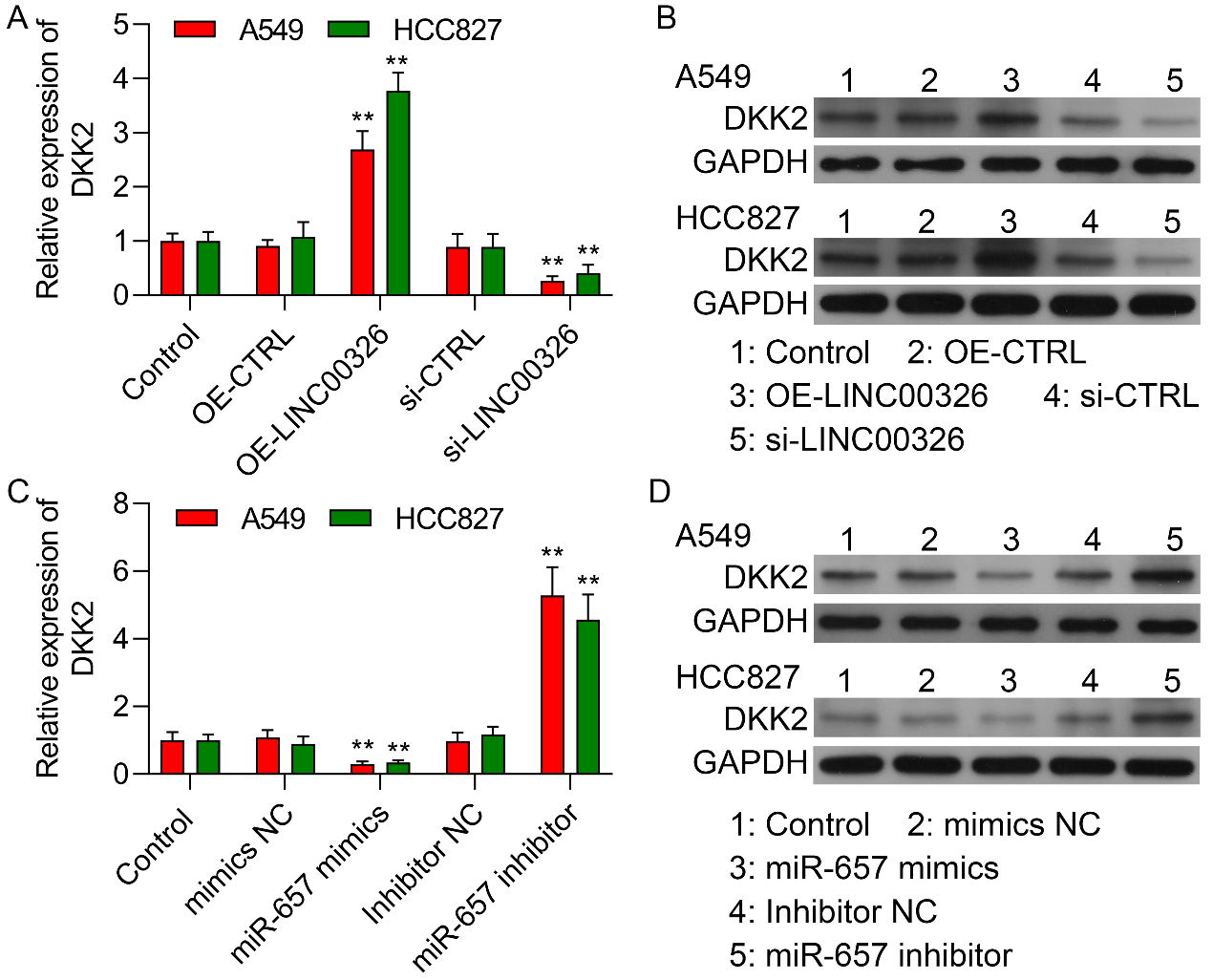


**Figure S4. (A-B)** Levels of DKK2 mRNA and protein in HCC827 and A549 cells transfected with OE-CTRL, OE-LINC00326, si-CTRL, or si-LINC00326, evaluated by RT-qPCR and western blotting. **(C-D)** DKK2 mRNA and protein levels in cells transfected with Control, miR-657 mimics NC, miR-657 mimics, miR-657 inhibitor NC, and miR-657 inhibitor vectors. Data are presented as mean ± standard deviation. The experiments were repeated three times. ** P < 0.01.


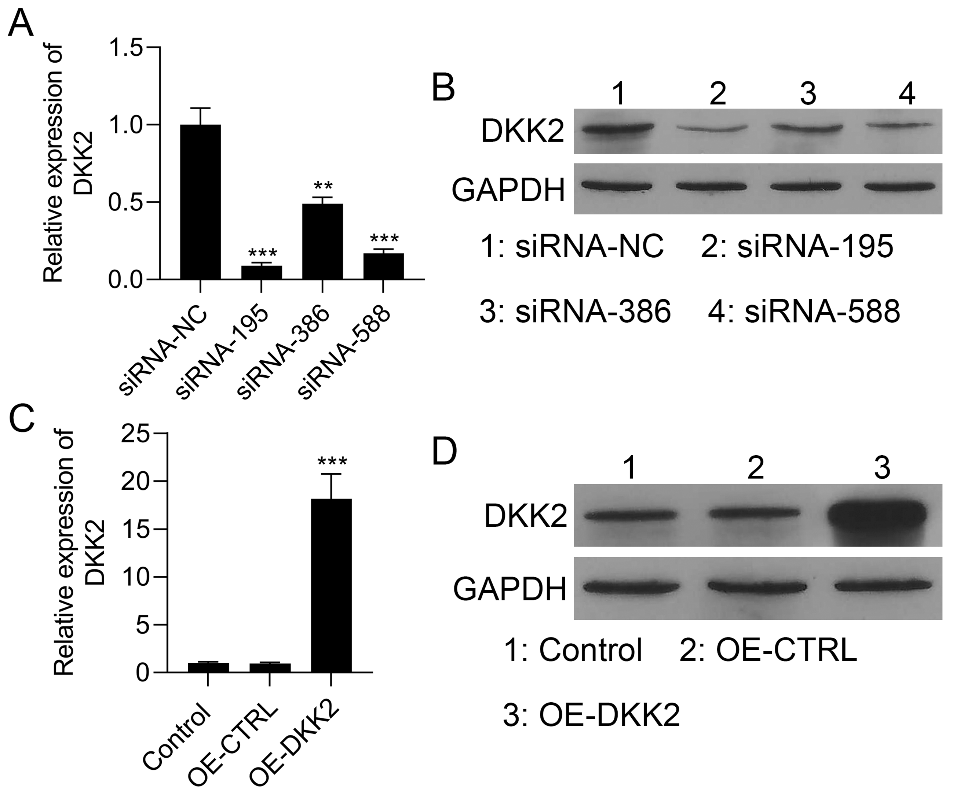


**Figure S5.** **(A-B)** Reduced DKK2 levels after DKK2 silencing, shown by RT-qPCR and western blotting analysis. **(C-D)** DKK2 levels in control and DKK2-overexpressing cells, shown by RT-qPCR and western blotting. **P < 0.01; ***P < 0.001.


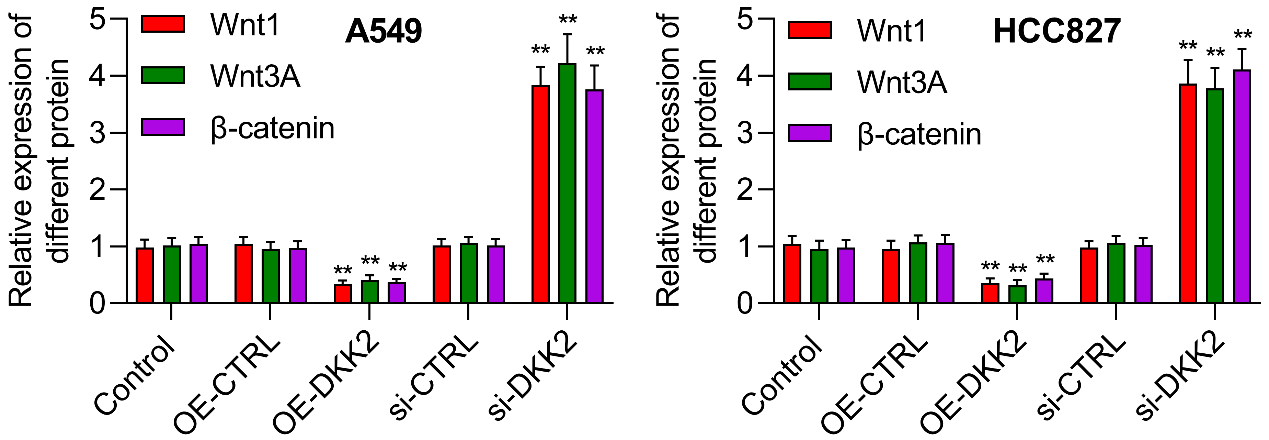


**Figure S6.** **Quantification of different protein expression levels.** Overexpression of DKK2 suppressed Wnt1, Wnt3A and β-catenin protein expression, while knockdown of DKK2 induced Wnt1, Wnt3A and β-catenin protein expression. **P < 0.01.


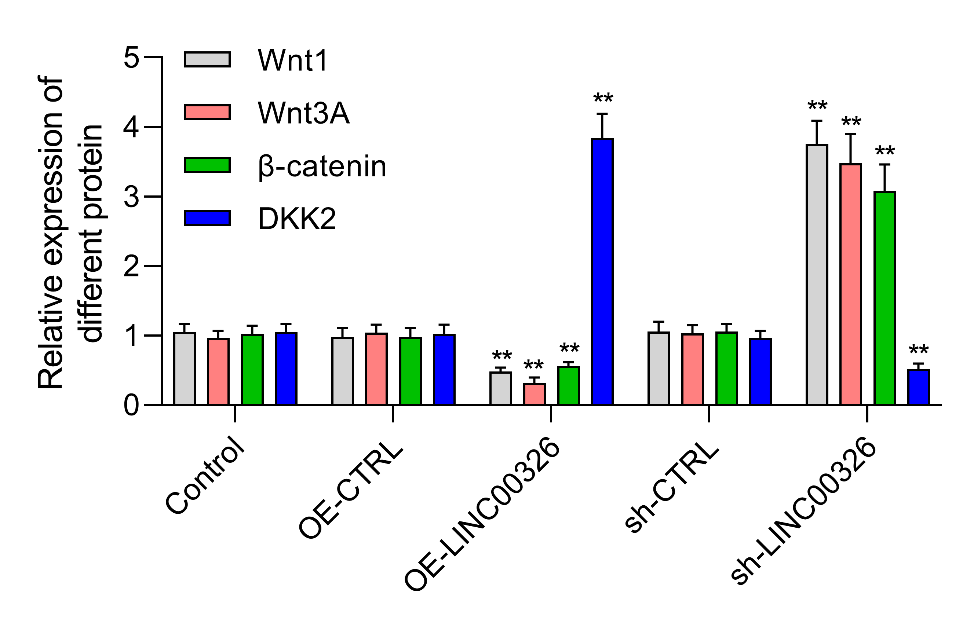


**Figure S7. Quantification of different protein expression levels.** Overexpression of LINC00326 promoted DKK2 expression and suppressed Wnt1, Wnt3A and β-catenin protein expression, while knockdown of LINC00326 suppressed DKK2 expression and induced Wnt1, Wnt3A and β-catenin protein expression. **P < 0.01.
